# Sustainable software development in science – insights from 20 years of Vanted

**DOI:** 10.1515/jib-2025-0007

**Published:** 2025-07-01

**Authors:** Falk Schreiber, Tobias Czauderna, Dimitar Garkov, Niklas Gröne, Karsten Klein, Matthias Lange, Uwe Scholz, Björn Sommer

**Affiliations:** Department of Computer and Information Science, University of Konstanz, Konstanz, Germany; Faculty of Information Technology, Monash University, Clayton, Australia; Faculty of Applied Computer Sciences and Biosciences, University of Applied Sciences Mittweida, Mittweida, Germany; Leibniz Institute of Plant Genetics and Crop Plant Research (IPK) Gatersleben, Seeland, Germany; Royal College of Art, London, UK

**Keywords:** tools, sustainable software development, network analysis and visualisation

## Abstract

Sustainable software development requires the software to remain accessible and maintainable over long time. This is particularly challenging in a scientific context. For example, fewer than one third of tools and platforms for biological network representation, analysis, and visualisation have been available and supported over a period of 15 years. One of those tools is Vanted, which has been developed and actively supported over the past 20 years. In this work, we discuss sustainable software development in science and investigate which software tools for biological network representation, analysis, and visualisation are maintained over a period of at least 15 years. With Vanted as a case study, we highlight five key insights that we consider crucial for sustainable, long-term software development and software maintenance in science.

## 1 Introduction

Sustainable software development in science, that is long term development and maintenance of research software, is a challenging endeavour. It typically involves creating tools for data representation, integration, analysis, simulation, visualisation, and the communication of results for specific projects or research questions. At the same time, those tools should maintain accuracy, ensure reproducibility, provide ease of use and support long-term usability including proper licenses. For example, scientific results published in papers can often only be reproduced if the software used is still available and usable.

There are many software packages and workflows that have been developed to solve scientific questions, but few have successfully achieved long-term sustainability. There are several reasons for this, including that scientific projects and related software development are often only funded through short-term research grants, and that software maintenance does not significantly contribute to the career advancement of those involved. While the emphasis on publishing research papers remains strong and research software development is still often undervalued as a research activity, this issue was particularly problematic in the past. Other reasons are that there is often insufficient institutional support for long-term software maintenance, and that software is frequently developed by different research groups independently and without joining forces. As a result it is often difficult to create, maintain, improve, and use software effectively over the span of at least 15–20 years.

As example for sustainable and FAIR research software development, we will provide an overview of positive examples for software tools in the field of biological network representation, analysis, and visualisation that are still maintained after 15 years. Further, we will use Vanted (Visualisation and Analysis of Networks comprising Experimental Data) as a case study of software developed over the past 20 years. Vanted is a tool that was originally designed for the analysis and visualisation of biological networks and related data. Over the years, it has been developed into a software resource in various fields, particularly in systems biology, bioinformatics, and related disciplines. We will explore how Vanted is a model of sustainable software development in science, and investigate its design principles, development, community involvement, and adaptability to meet the changing needs of its user base. We will share the lessons learned, offering guidelines for sustainable software development.

The topic of this paper aligns very well with the 20th anniversary of the *Journal of Integrative Bioinformatics* (and this special issue dedicated to this event), in particular as: (1) the journal promotes software accessibility since its inception and provides with JIB.tools an environment for that [1], 2], (2) integrative bioinformatics is an important aspect of the Vanted development, for example, by novel ways for data integration, and (3) Vanted itself is as old as the journal.

## 2 Evolution of software development

Vanted incorporates key concepts of modern software development as outlined at the end of this section. Here we will give a very brief overview of the evolution of software development, which has been driven by technological advances, growing complexity in hardware and software, and changing user needs. Further, scientific software development progressed in parallel to, and was influenced by, the transition of pen and paper science to initial implementations and then to tools and libraries to provide software for reproduction and replicability [3].

Software development has been changed over time, and each phase can be characterised by specific challenges and methodologies:

The 1940s: Software development started when the first programmable computers were invented. At that time, software and hardware were not really considered distinct parts, as programming was closely tied to the physical design of the computers. Early universally programmable computers such as Zuse Z3 (1941) [4], ENIAC (1943–1946) [5], and UNIVAC I (1951) [6] were programmed low level using machine-specific code, therefore programs could not be easily transferred between different machines.

The 1950s: First high-level programming languages such as Fortran for scientific and engineering calculations (started 1953, first implementation 1957) and LISP for symbolic computation and (early) artificial intelligence research (started 1958, first implementation 1960) were developed. Those languages helped separating hardware from software, enabling programs to be written independently of the underlying computer architecture, and therefore making software development more general and improving portability across different computer systems.

The 1960s and 1970s: Due to increasing software complexity, the focus shifted towards improving software design and development methodologies, such as the imperative programming paradigm. This included declarative programming which emphasised creating programs in ways that were easier to write, understand, debug, and maintain. Moreover, object-oriented programming emerged as a paradigm that organises code into objects, which encapsulate both data and behaviour. The object-oriented approach has had significant impact on the development of modern software, improving modularity, code reusability, and maintainability. The term software engineering was already coined in the 1960s [7], but it was only later that this field gained wider influence.

The 1980s and 1990s: There was an increasing need for more formal software engineering practices to deal with issues such as software project management, platform independence, quality assurance, and scalability. Therefore, the concept of the software development lifecycle was developed. The waterfall model, one of the first formalised models, was introduced by Winston Royce in 1970 [8]. Although even Royce himself commented that it had major problems as testing only happened at the end of the process, it became a standard approach for many software projects. The problems of the waterfall model later led to the development of iterative and incremental models of software development such as Agile software development [9]. While approaches and frameworks such as Agile and Scrum gained widespread adoption, the “Agile is dead” discussion during the 2020s highlights ongoing debates about their relevance and effectiveness. More generally, many practices designed for commercial software development cannot be directly transferred to academic environments, which face unique challenges and aims.

The 2000s: The new millennium brought a great availability of open-source software. This changed the landscape of software development, enabling easier collaboration, reuse of code, and joint software development. Linux, the computer operating system created by Linus Torvalds and first released in 1991, is a prominent success story of the open-source movement. An important aspect of open-source software development is the community-driven development, where users and developers actively contribute to improve the software.

As we will discuss in more detail later, Vanted incorporates key concepts of modern software development:

– Vanted is built on an *object-oriented approach* (using Java) that promotes code reusability, modularity, and maintainability.
– Its development follows an *iterative and incremental software engineering* model that makes it easy to adapt to new projects and challenges.
– The software is *open-source* to promote free use and community-driven development.

## 3 Rules and recommendations for good software development in science

What defines good modern software development, in particular the development of research software in science? How can flexibility, collaboration, and continuous improvement be fostered, and how can proper, maintainable code and an user-centric design be promoted? A wealth of literature exists on this topic, including guidelines for software development in science, such as the *Ten Simple Rules for the Open Development of Scientific Software* [10] which, in short, state:

1. Rule: Don’t Reinvent the Wheel
2. Rule: Code Well
3. Rule: Be Your Own User
4. Rule: Be Transparent
5. Rule: Be Simple
6. Rule: Don’t Be a Perfectionist
7. Rule: Nurture and Grow Your Community
8. Rule: Promote Your Project
9. Rule: Find Sponsors
10. Rule: Science Counts

Recently, the FAIR4RS initiative has introduced the FAIR principles for research software [11] in an attempt to tackle some of the challenges underlying current research software. In the context of research software FAIRification, the R set of principles advises software and its components to be usable and reusable. It is evident that research software management differs from research data management [11]. For research software to remain (re-)usable over time, it should be designed, implemented, and maintained as such continuously from the start.

While we concur with those guidelines, there are still crucial aspects that require greater attention, particularly concerning software sustainability over extended periods (15–20 years). Based on our two decades of experience developing Vanted, we would like to highlight the following additional recommendations:

1. Recommendation: Long-Term Design and Adaptability
2. Recommendation: Continuous Contribution to Science: Research in the Application Domain(s)
3. Recommendation: Continuous Contribution to Science: Research in the Computer Science Domain
4. Recommendation: Open-Source Nature and Community Contribution
5. Recommendation: User-Centric Development and Support

Before discussing the details of our five recommendations, we will first provide an example of software sustainability in practice by examining tools for biological network representation, analysis and visualisation over the past 15 years in Section 4. Next, in Section 5, we will give an overview of the Vanted system, its architecture and its usage. We will present the five recommendations in Sections 6–10, supported by examples from the development of the Vanted software. We conclude with Section 11 to discuss our findings, outline further Vanted developments and present more details of the connection between 20 years of Vanted and to the 20 years of the *Journal of Integrative Bioinformatics*.

## 4 The reality of software sustainability – an example from biological network analysis and visualisation

In 2009, some of the authors conducted an unpublished study of available tools for biological network representation, analysis, and visualisation based on an extensive literature and web search. This collection was based on aspects such as data exchange (importing/exporting networks), dynamic visualisation (layout of nodes and edges, editing capabilities), visualisation elements (data representation using colour-coding, variations in node sizes or edge thickness), and data analysis (network analysis, statistics). The aim in 2009 was to extend the scope of earlier work by Pavlopoulos et al. [12] which tried to cover a broad range of use cases and focused on a few tools, a study by Saraiya et al. from 2005 [13], as well as a comparative study published by Suderman and Hallett in 2007 [14] which studied nearly 40 network visualisation tools. Please note that for certain tools, references to papers published after 2009 have been included when the tool was available online but no publication existed in 2009. Additionally, this collection reflects our knowledge as of 2009; a contemporary collection would include some different tools while omitting others.

During this study, 174 relevant tools were identified and investigated further. 51 out of these 174 tools were open-source, 24 commercial, 13 were web-based, and 39 tools mentioned in the literature were already no longer available on their respective websites in 2009. The tools with the most citations that time were DAVID [15] (818 citations), GeneVestigator [16] (738 citations), and Cytoscape [17] (618 citations). DAVID and GeneVestigator were well established in the scientific community and widely used for retrieval of information about microarray data, which explains the high citation count at the time. Cytoscape was primarily used for network analysis and visualisation, gaining traction as the field of systems biology expanded.

After 15 years, in 2024, we reviewed the status of the initially catalogued tools, see Table 1. The result of this investigation is as follows: 89 of the tools identified in the 2009 data collection (approximately 51 %) are no longer available. We categorize a tool as being “no longer available” if it is no longer accessible as defined in its first publication or the source code repository or download URL is no longer resolvable. Specifically, for none of these tools, even outdated or unsupported versions remain accessible from their developers. It should be noted that 36 tools (around 21 %) were already unavailable in 2009 (published before 2009 but not available in 2009). Therefore, 53 of the tools listed and still available in 2009 have become inaccessible over the last 15 years. Furthermore, 35 tools (around 20 % of all tools) can be classified as obsolete, as they are available, but have not received any updates in the last five years. In Table 1, we indicate obsolete tools with a dagger symbol followed by the exact year the last update was carried out. Of these 35 tools, 30 have remained without updates for (more than) a decade. This classification is based on evidence from release histories, website activity, copyright notices, and associated publications.

**Table 1:** Overview of tools.

Tool name	Year first publication	Ref.	Still available?
A-cell	2001	[18]	No
aiSee	n/a	[19]	No
Advanced pathway painter	2003	[20]	Yes
Arcadia	2008	[21]	Yes (†2014)
ArrayXPath	2004	[22]	No
Atlas	2005	[23]	Yes
AVIS	2007	[24]	No
Biological concept diagram editor	2008	[25]	No
BINViz	2008	[26]	Yes (†2013)
Bio sketch pad	2001	[27]	No
BioBiblioMetrics	2000	[28]	No
BioCarta	n/a	[29]	No
Biogranat	2008	[30]	Yes (†2014)
BioGraphNet	2004	[31]	Yes (†2013)
BioJAKE	1999	[32]	No
BioLayout	2000	[33]	Yes
BiologicalNetworks	2005	[34]	No
BioMiner	2002	[35]	No
BioPath	2002	[36]	No
BioPathwise	2007	[37]	No
BioPax	2003	[38]	Yes
BioPP	2007	[39]	No
BioSpice	2002	[40]	Yes (†2012)
BioTapestry	2005	[41]	Yes
BioUML	2002	[42]	Yes
BNArray	2006	[43]	Yes (†2006)
CADLIVE	2003	[44]	Yes (†2010)
Cell illustrator	2004	[45]	Yes
CellDesigner	2003	[46]	Yes
CellNetAnalyzer	2003	[47]	Yes
CentiBin	2006	[48]	Yes (†2011)
Pathway tool software	2005	[49]	No
COB editor	2005	[50]	Yes (†2005)
CPN tools	1999	[51]	Yes
Cell system markup language	2000	[52]	No
CUtenet	2000	[53]	No
Cyclone	2005	[54]	Yes (†2013)
Cytoscape	2003	[17]	Yes
DAVID	2003	[15]	Yes
DBmcmc	2003	[55]	No
Dynamic signaling maps	n/a	[56]	No
E-cell	1999	[57]	Yes
EMMA2	2008	[58]	Yes (†2013)
Edinburgh pathway editor	2006	[59]	Yes (†2013)
ExPASy	2003	[60]	Yes
ExPlain	2006	[61]	Yes
GENAW	n/a	[62]	No
GeneGobi	2004	[63]	No
GenePath	2003	[64]	Yes
GeneScene visualizer	2005	[65]	No
GeneSpring GX	2008	[66]	Yes
Genetic network analyzer	2002	[67]	Yes
GeneVestigator	2004	[16]	Yes
GeneView	2007	[68]	No
GeneWays	2003	[69]	No
GenMAPP	2005	[70]	No
GenoLink	2006	[71]	No
Genome3DExplorer	2005	[72]	No
Genomic object net	2003	[73]	No
GEOMI	2006	[74]	No
Gepasi	1989	[75]	Yes (†2002)
GEPAT	2007	[76]	Yes (†2013)
GeXpert	2006	[77]	Yes (†2013)
GlycoBrowser	2008	[78]	No
GoMiner	2003	[79]	No
Graphlet	1999	[80]	No
GraphViz	2003	[81]	Yes
Gravisto	2004	[82]	Yes
Grid cellware	2004	[83]	No
GridLayout	2005	[84]	No
GSCope	2006	[85]	No
IM browser	2006	[86]	Yes (†2012)
InNetics PathwayLab	n/a	[87]	No
INOH: pathways and ontologies	2003	[88]	No
IntAct	2004	[89]	Yes
Interviewer	2005	[90]	Yes (†2004)
Ingenuity pathways analysis	2004	[91]	Yes
J2dPathway	2008	[92]	Yes (†2016)
Jarnac	2000	[93]	Yes (†2012)
JDesigner	2002	[40]	Yes (†2012)
jSquid	2008	[94]	Yes (†2008)
KappaView	2005	[95]	Yes (†2016)
KEGGanim	2007	[96]	Yes
KGML-ED	2007	[97]	No
KGraphViewer	2007	[98]	Yes
Kinetikit	2003	[99]	Yes (†2005)
KnowledgeEditor	2002	[100]	No
MapMan	2004	[101]	Yes (†2013)
MAPPFinder	2003	[102]	No
MARGBench	1999	[103]	No
Mavisto	2005	[104]	Yes
megNet	2005	[105]	No
Metabolic IsaViz	2005	[106]	Yes (†2007)
MetaCore	n/a	[107]	Yes
EcoCyc	2002	[108]	Yes
MetaNetter	2008	[109]	Yes
MetaReg	2008	[110]	Yes (†2008)
metaSHARK	2006	[111]	No
MetNet3D	2005	[112]	No
MicroarrayDB	2005	[113]	No
Metabolic network visualizer	2003	[114]	No
MOVE	2006	[115]	No
Narrator – a graph-based modelling tool	2007	[116]	Yes (†2016)
NeAT	2008	[117]	No
NetBuilder	2002	[118]	No
Nodes3D	2006	[119]	No
ONDEX	2005	[120]	Yes (†2019)
ontoTools	2007	[121]	Yes (†2012)
Osprey	2003	[122]	No
Pajek	1998	[123]	Yes
PathArt	n/a	[124]	No
PathBank	2006	[125]	No
PathBuilder	2007	[126]	No
PathCase	2003	[127]	No
PathDB	2000	[128]	No
PathFinder	2002	[129]	No
PathMAPA	2003	[130]	No
pathSCOUT	2003	[131]	No
PathVisio	2008	[132]	Yes
Pathway analytics	2007	[133]	No
Pathway assist	2003	[134]	No
Pathway builder 2.0	2005	[135]	Yes
Pathway builder	2005	[136]	No
Pathway processor	2002	[137]	No
Pathway studio	2003	[134]	No
Pathway tools	2002	[138]	Yes
PathwayLab	2009	[139]	No
PathwayLogic	2002	[140]	Yes
Patika	2002	[141]	No
PaVESy	2004	[142]	No
Pathway hunter tool	2005	[143]	Yes
PhyloGrapher	2001	[144]	Yes (†2003)
PIMWalker	2005	[145]	No
PIVOT	2003	[146]	Yes (†2003)
PNE – pathway network editor	2007	[59]	Yes (†2014)
Prefuse	2005	[147]	Yes
ProcessDB	2001	[148]	Yes
ProMoT	2003	[149]	No
ProteoLens	2008	[150]	Yes
PROTON	n/a	[151]	No
ProViz	2005	[152]	No
PubGene	2000	[153]	Yes
PWComp	2002	[154]	No
pyNetConv	2005	[155]	Yes (†2013)
QPACA	2006	[156]	No
Reactome	2005	[157]	Yes
ROSPath	2004	[158]	No
SBW	2003	[159]	Yes
SHARKview	2007	[160]	No
SHriMP	2004	[161]	No
simBio	2006	[162]	No
SimWiz	2004	[163]	No
Snoopy	2000	[164]	Yes
Sofia	2008	[165]	No
Spike	2008	[166]	Yes
SYCAMORE	2008	[167]	No
Teranode design suite	2008	[168]	No
BioPath	2001	[169]	No
Tom sawyer	2009	[170]	Yes
ToPNet	2004	[171]	No
Pathway editor	2005	[172]	No
Unipath	2003	[173]	No
Vanted	2006	[174]	Yes
Vector PathBlazer	n/a	[175]	No
VisANT	2004	[176]	No
VitaPad	2005	[177]	Yes (†2013)
WebGestalt	2005	[178]	Yes
WebInterViewer	2003	[179]	No
WikiPathways	2008	[180]	Yes
WilmaScope	2002	[181]	Yes (†2013)
YANAsquare	2007	[182]	Yes
yFiles	2001	[183]	Yes

Importantly, with 48 tools, less than a third of all initial tools (about 28 %) are actively available or show no clear evidence of being unsupported. Here, “actively available” is defined as having undergone at least one update within the past five years. “No clear evidence of being unsupported” indicates that while recent updates could not be confirmed due to limited information, a functional download link is accessible. That means that the majority of tools is not available anymore. This has profound impact, for example, relating to the reproducibility of scientific studies conducted using those tools.

## 5 Vanted – a brief overview

As mentioned, Vanted is a tool for the exploration, analysis and visualisation of biological networks and related experimental data. The development of Vanted started in 2003/2004, with its first publication in 2006 [174], and version Vanted v2 [184] with significant changes to core plug-ins has been released in 2012. It should be noted that the underlying system is even older: Vanted is based on Gravisto [82], a general graph editing and layout system, which was already in development end of the 1990s. From Gravisto the Vanted software inherited a flexible and modular concept of plug-ins. Internally every major part of Vanted is a plug-in, and as of Vanted v2.8.8, there are 91 plug-ins in the core. Besides user interface and core framework functionality, plug-ins are organised into several sub-domains: (1) specialised import and export support, (2) database access, (3) network layout algorithms, and (4) other algorithms. Plug-ins for import and export include support for SBML [185], BioPAX [38], SBGN-ML [186], KGML (the exchange format for KEGG pathway maps) [187], GraphML [188], and GML [189] (Vanted’s default network format). Among the extensions for database access are plug-ins for KEGG [187], BioModels [190], MetaCrop [191], and RIMAS [192].

**Figure 1:**
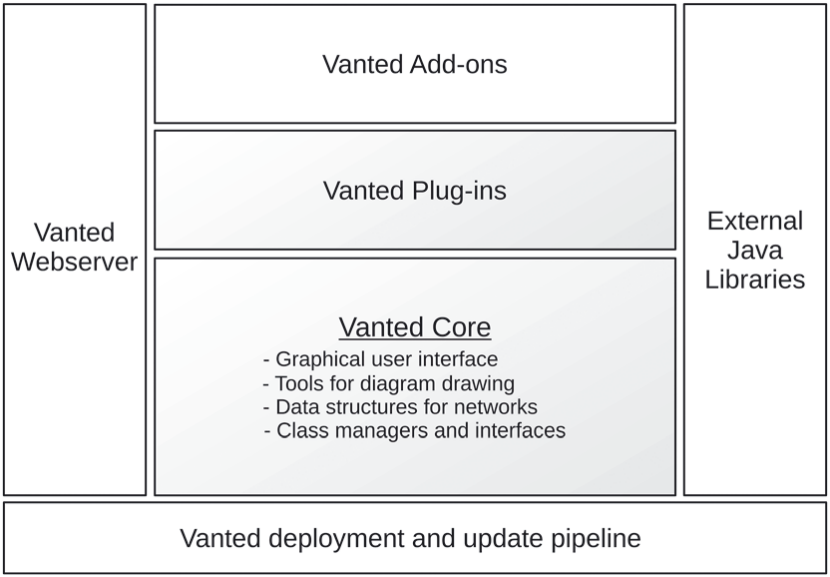
The architecture of Vanted consists of several classes of interfacing components. The core (shaded area) is responsible for overall architecture (class managers and interfaces), for network data structures, for diagram drawing and for the user interface. The core also consists of a number of plug-ins providing dedicated functionality. On top of the core, users write add-ons to extend for specific functionality. Java libraries provide external tools and interfacing with formats and standards. The core communicates with Vanted’s webserver to find updates. Any updates are deployed to the webserver through the deployment and update pipeline.

See Figure 1 for an overview of Vanted’s architecture. Vanted comprises a modular architecture, capable of supporting a range of different use cases and scenarios. In addition to the core’s internal plug-ins, external plug-ins, so called add-ons, are also part of the architecture. Vanted’s add-ons provide the necessary degree of customisation and extensibility to the core functionality, while assuring a proper separation of concerns. This concept has been important in the long-term maintenance of Vanted, such that Vanted’s core includes only the functionality which should be actively maintained and made compatible with past and new versions of its core, add-ons, and, of course, Java itself.

Developed using Java, Vanted embodies Java’s “*write once, run everywhere*” philosophy, enabling it to run on all three major operating systems with minimal additional setup. To install and run Vanted on a desktop, users need to have the Java Runtime Environment (JRE, available at www.java.com/download) pre-installed. To download and install Vanted, a user can simply download the latest version from the Vanted website [193].

Vanted is fully open-source, available on GitHub under the GPL-2.0 license. On GitHub, developers can also find a wiki and users can report issues. Add-on developers can use a so-called example add-on as a template. Tutorials, examples and further documentation are also available on the Vanted website.

## 6 Recommendation 1: long-term design and adaptability

Make the software modular and continuously adaptable from the beginning.

An important aspect of sustainable software development in science is creating systems that are flexible and adaptable enough to meet the (changing) demands of users while maintaining stability, reliability, and performance. A system should be modular to enable updates, adaptions, and additions of new functionalities without disrupting existing components. Vanted fulfils these principles through several design features:

– Modular architecture: Internally, every major part of Vanted is a plug-in, which can be dynamically changed or replaced by new code. For example, different views of a network, such as a view “node-link diagram” or a view “statistical properties of the network” are implemented as plug-ins, being easily exchangeable. Another example are different algorithms, which are also implemented as separate plug-ins.
– Extensible architecture: Vanted provides the concept of add-ons, external “plug-ins” which can provide new functionality without the need to change internal (core) functions and allow to add new algorithms, data types, visualisation techniques and similar without disrupting the core functionality.
– Scalable performance: Leveraging the concept of plug-ins allows the core functionality to remain efficient even as datasets grow. For instance, visualising a network as a node-link diagram can be resource-intensive when dealing with networks (graphs) containing tens of thousands of nodes and edges with related data shown. The plug-in approach enables switching to a less resource-demanding view, for example, displaying only key information for large networks (such as statistical parameters) instead of all elements of the entire network.
– Object-oriented approach: Using Java Vanted is based on an object-oriented approach that promotes code reusability, modularity, and maintainability in the long run.

This flexibility ensures that Vanted remains relevant as new scientific challenges and data types emerge. Several add-ons have been developed to extend the functionality, for example, FluxMap [194] for flux visualisation, PetriNets [195] to allow the simulation of Petri nets, CentiBiN [196] which extends the software to investigate different centrality measures in networks, FBA-SimViz [197] for interactive visualisation of constraint-based metabolic models, and SBGN-ED [198] for editing, validating, and translating of SBGN maps. Add-ons also offer a great way to encourage external (community) participation and enable the software to be used in teaching, such as allowing students to develop new functionality as part of their project.

However, we also learned that modifying the core system, as it was done for various reasons from version 1 to version 2 in 2012, introduced challenges. Due to changes in both the underlying Java system and related external libraries in version 2, not all add-ons remained compatible with the new Vanted version. However, separating functionality into core and add-ons allows us to prioritise resources to maintaining the core, while less relevant or outdated add-ons can be phased out over time.

## 7 Recommendation 2: continuous contribution to science: research in the application domain(s)

Make the software contributing to science – in the application area(s).

A key aspect of Vanted’s sustainability is its continued impact on scientific research. Over the past 20 years, the software has made significant contributions to the field of life sciences, enabling the visualisation and analysis of complex biological networks with related experimental data that were otherwise difficult to interpret. By facilitating the exploration of these networks, Vanted has helped researchers uncover new insights into cellular processes, disease mechanisms, and other important biological phenomena.

From our experience, it is crucial that scientific software is developed in continuous and close collaboration with partners from the application domain(s). This approach has been central to Vanted’s development, ensuring that it meets the needs of its diverse user base. Examples of such joint projects include plant sciences, where Vanted was initially developed in collaboration with plant scientists, e.g., [199], 200], animal sciences [201], medicine [202], 203], and microbiology [204], 205]. Additionally, Vanted has been used to build and graphically enhance with interactive visualisations biological databases such as MetaCrop [206], Rimas [192], and QSDB [207]. These collaborations have consistently brought new ideas and methods for the ongoing development of Vanted.

Vanted has been utilised in numerous external projects and publications, where it has often been explicitly cited; however, there are also instances where it is merely acknowledged or not mentioned at all despite its use. Examples from biology include its use in detailing the lipid composition in pollen [208], where it was applied to analyse and visualise the synthesis and breakdown of lipids, its contribution by means of statistical and visual analyses to the investigation of combined abiotic and biotic stress factors in plants [209], its use to analyse the central metabolism in developing oilseeds [210], and for the visualisation of flux and transcript data [211]. Examples from medicine, just one of the other application areas, include its use in the visual analysis of metabolites for their role in PD-1 blockade therapy in cancer research [212], to explore gut microbe metabolites in Alzheimer’s disease research [213], and to identify drug targets in Covid-19 disease mechanisms [214]. In visualising omics data, Vanted has been known to support “interactive editing particularly well” [215].

## 8 Recommendation 3: continuous contribution to science: research in the computer science domain

Make the software contributing to science – in computer science.

In addition to the application areas, Vanted has been used to drive novel methodological developments in computer science, in particular in the fields of network visualisation and topological network analysis.

Network layout methods are important for visual network exploration, and Vanted provides several well-known methods such as force-directed, stress minimisation, multi-level, grid and tree layout. However, existing network layout methods are often insufficient [216]. For example, hierarchical network representations (such as clustered graphs or networks of pathways) and their interactive exploration are often relevant for biological networks. Different novel approaches have been developed and implemented in the Vanted framework, already starting in 2007 with the dynamic exploration and editing of KEGG pathway diagrams [97]. To help preserve the mental-map [217] during the exploration of networks with clusters or sub-networks, Vanted provides methods which have been developed over the years. For example, NetPartVis to visualise non-overlapping clusters or partitions of graphs by laying out overview graph and sub-graphs (partitions) in a coordinated, mental-map preserving way [218], using glyphs, brushing, and topological information of the related pathways for interactive visualisation [219] as well as a decomposition method which is part of the LMME add-on [220]. We also investigated group-based visual transformations such as de-emphasising groups by opacity, position or size, aggregating groups and hiding groups to find the most suitable approach for the exploration of networks with clusters or sub-networks [221].

For the analysis of biological networks novel algorithms have been developed and usually implemented in Vanted, such as flux-based centrality analysis [222], motif-based centrality analysis [223], and pattern detection under different frequency concepts for the analysis of motifs in networks [224].

More recently, Vanted has been also used for analysing networks in other domains, for example, to investigate the relationship between Celtic knots and specific graphs [225].

These examples underscore the critical role of scientific contributions, such as new network analysis algorithms and novel visualisation methods, in pushing the boundaries of computer science during sustainable research software development. There has to be scientific progress in the application domain(s), but also in the computer science domain.

## 9 Recommendation 4: open-source nature and community contribution

Make the software open-source and support community involvement.

There are several advantages of open source software and community involvement, and the open-source nature of Vanted has been important for its longevity:

– By making the software freely available and open for modification, researchers around the world can contribute improvements and bug fixes. This has helped build a diverse community of users and developers who contribute to the software’s enhancement. Examples include usage and extension independent of our team as done for the visualisations presented by Yugi et al. [226], as well as joint development of add-ons, for example, with the group of Andreas Kerren for glyph-based navigation of metabolic networks [219].
– Our group has moved several times over the past 20 years including between different countries, as Vanted’s development sustained. Having the code open source has made it easier to transfer the code between different institutions or universities without facing licensing issues.
– The open-source model ensures that Vanted can be integrated into a variety of workflows and customised to meet the specific needs of different research fields. An example is the inclusion of Vanted (and its SBGN-ED add-on [198]) into the ecosystem of the Covid-19 disease map community [227].

The ability to customise and adapt the software freely is a major advantage for long-term usability and maintainability. Vanted has embraced the concept of community-driven development, where users not only benefit from the software, but also contribute to its evolution, providing a sustainable cycle of usage, feedback, and improvement. A community-driven approach is crucial for the long-term support of software; it is a process that needs a low entry barrier to build an initial user base and then create further momentum, such as through events or other incentives.

## 10 Recommendation 5: user-centric development and support

Make the software user-friendly, also for non-expert users.

The development of Vanted was strongly driven by user needs, adapting the software based on feedback from both researchers and developers. This user-centric approach has helped that Vanted remains a relevant tool as new scientific challenges emerge. In particular, the use in collaborative projects has ensured that Vanted evolved in a way that addresses the real-world problems faced by its users. Early interdisciplinary work for the representation of experimental biological data in metabolic networks [228] and for analysing the topology of such networks led to prototypes called DBE-Gravisto [229], PatternGravisto [230] and MAVisto [104]. These early prototypes were instrumental for the successful development of Vanted which was directly based on experience with those prototypes and their early application to scientific questions such as presented by Rolletschek et al. [231]. Functionality of these early prototypes proved so useful in collaborative projects that it was later included into Vanted as plug-ins and add-ons. This user-focused collaboration continued over the years, recent examples of add-ons from interdisciplinary collaborations are PathwayNexus [232] for interactive metabolic data analysis together with the group of Marcel Leist, and for the layout of anatomical structures and blood vessels based on the foundational model of anatomy [233] together with Bernard de Bono.

Sustainability in scientific software development is also supported by comprehensive documentation. The Vanted developers have provided user guides, (video) tutorials and documentation that help users understand how to use the software effectively, even if they are new to bioinformatics and biological data analysis. There are also protocols and tutorials (e.g., [234], 235]) which provide guidelines and step-by-step training, and we provided several workshops and in-class tutorials to support users making their first steps with the tool and analysing their own data. The production of documentation and tutorials is time-intensive, but is important for onboarding new users and ensuring that users can take full advantage of the software.

In addition, it is often necessary to not only provide a tool but some broader ecosystem offering additional functionality necessary for data integration and analysis. For example, for Vanted this has included

– ways to store experimental data such as DBE2 for the management of experimental data [236],
– databases providing biological pathways such as MetaCrop [237] and RIMAS [192],
– connections to other databases with experimental data such as OPTIMAS-DW [238], and
– services to predict database links in biomedical databases [239], 240].

## 11 Conclusions

The development of Vanted over the past two decades is an example of how research software can be developed to achieve long-term sustainability. Its modular architecture, adaptability, open-source nature and community-driven development have allowed it to thrive and remain an important tool for researchers. This led to five insights that we consider crucial for sustainable, long-term software development and software maintenance in science:

– Recommendation 1: Long-Term Design and Adaptability
– Recommendation 2: Continuous Contribution to Science: Research in the Application Domain(s)
– Recommendation 3: Continuous Contribution to Science: Research in the Computer Science Domain
– Recommendation 4: Open-Source Nature and Community Contribution
– Recommendation 5: User-Centric Development and Support

Vanted is one of the tools for biological network analysis and visualisation still available and maintained after 15 years, and only roughly one third of the initially developed and published tools fall in this category.

We plan to develop Vanted further in the future. We like to improve the inclusion of three-dimensional (3D) data, both 3D network data and spatial information. The importance of 3D in network visualisation has already been discussed many years ago [241], 242], and a Vanted-based version for early data integration of 3D data that allowed creating views on integrated multi-domain data including 2D, 3D and network data has been made available already in 2011 [243]. Newer developments based on the raise of Immersive Analytics [244], 245] include the integration and virtual reality exploration of biomedical data [246] and the support of different display modalities such as transitional or hybrid interfaces in Vanted [247], 248]. Here, we plan to further extend this, for example, by including network-based information (such as signal transduction and gene regulatory networks) in our 3D based work for spatial transcriptomics [249]. We also started to investigate suitable network visualisation approaches in 2D, 2.5D (see e.g., [250]) and 3D [251] and plan to further investigate this and include useful approaches, for example, as Vanted add-ons. Another direction of future developments is a better support for constraint-based layout algorithms to provide more precise and customisable visual representations of complex networks while preserving structural and relational context (i.e., the user’s mental map).

The focus of the *Journal of Integrative Bioinformatics* over the last 20 years is also related to the development of the Vanted framework. Since its inception, the journal has supported software accessibility, offering an environment for this purpose through JIB.tools, and Vanted provides free accessibility and supports long term maintenance. In addition, integrative bioinformatics, the key topic of the journal, is an important aspect of the Vanted development, for example, by novel ways for data integration. The *Journal of Integrative Bioinformatics* is also very committed to support the development of standards in systems and synthetic biology; special issues focusing on COMBINE [252] standards have been released regularly, offering updates since 2015 [253]. We believe that standards are very important, and Vanted not only supports several COMBINE standards such as SBGN, SBML and BioPax, but it is also a reference implementation for the SBGN [254] standard, supporting all three graphical languages (Process Description [255], Entity Relationship [256] and Activity Flow [257]) and providing additional functionality such as editing and validating SBGN maps [198], conversion of KEGG metabolic pathways to SBGN maps including automatic layout [258] and the translation of SBGN maps from Process Description to Activity Flow [259].

Sustainable software development in science, as demonstrated by the Vanted system, ensures tools not only remain relevant and functional over decades, but also continue to evolve. This enables researchers to address new challenges and fosters enduring contributions to scientific discovery.
